# Modeling the Impact of Sexual Networks in the Transmission of *Monkeypox virus* Among Gay, Bisexual, and Other Men Who Have Sex with Men — United States, 2022

**DOI:** 10.15585/mmwr.mm7135e2

**Published:** 2022-09-02

**Authors:** Ian H. Spicknall, Emily D. Pollock, Patrick A. Clay, Alexandra M. Oster, Kelly Charniga, Nina Masters, Yoshinori J. Nakazawa, Gabriel Rainisch, Adi V. Gundlapalli, Thomas L. Gift

**Affiliations:** 1 CDC Monkeypox Emergency Response Team.

Transmission of *Monkeypox virus* (MPXV) during the 2022 multinational monkeypox outbreak has been associated with close contact, primarily sexual behavior, between men (*1*). Survey data suggest that gay, bisexual, and other men who have sex with men (MSM) have taken steps to protect themselves and their partners from monkeypox, including reducing one-time sexual partnerships (*2*). CDC simulated dynamic network models representing the sexual behavior between MSM. Men with more than one partner in the preceding 3 weeks had 1.8–6.9 times the risk for acquiring monkeypox as did men with only one partner. Although one-time partnerships represented <3% of the total daily partnerships and 16% of the sex between men on any given day, they accounted for approximately 50% of MPXV transmission. In this model, a 40% decrease in one-time partnerships yielded a 20%–31% reduction in the percentage of MSM infected and a delay in the spread of the outbreak. A decrease in one-time partnerships not only decreased the final percentage of MSM infected, but it also increased the number of days needed to reach a given level of infection in the population, allowing more time for vaccination efforts to reach susceptible persons. If decreasing one-time partnerships were combined with additional mitigation measures such as vaccination or shorter time from symptom onset to testing and treatment, this effect would be higher. Reductions in one-time partnerships, a change in behavior already being reported by MSM, might significantly reduce MPXV transmission.

CDC adapted previously developed models of sexual infection transmission used to study HIV and gonorrhea transmission in the United States* (*3*,*4*); this framework has also been used to study MPXV spread in Belgium (*5*) (Supplementary Box 1; https://stacks.cdc.gov/view/cdc/120605). In this dynamic network modeling framework, men may have zero or one main partnership at a time, assumed to last 477 days on average, as well as zero, one, or two casual partnerships at a time, assumed to last 166 days on average. Men may also form one-time partnerships that last 1 day, meant to mimic a single sexual encounter that is not repeated. A man could possibly have main, casual, and one-time partnerships concurrently. The model includes six strata of sexual activity, which differ in their rate of one-time partnership formation (Supplementary Box 2; https://stacks.cdc.gov/view/cdc/120606). These partnership strata were informed by data collected during 2016–2019 from MSM in Atlanta, Georgia, who reported the number, type, and duration of their current sexual partnerships (*3*,*4*,*6*).

MPXV natural history and MSM care-seeking behaviors were based on previous publications, and metrics observed during the current outbreak response when available (*7*) (Supplementary Box 2; https://stacks.cdc.gov/view/cdc/120606). Because of uncertainty about how widely MPXV might spread among MSM, two scenarios in which 10 highly active cases were introduced to a population of 10,000 MSM were simulated, representing lower and higher transmission, by adjusting the transmission probability per act, so that MPXV would eventually infect approximately 15% (lower transmission) and 25% (higher transmission) of MSM. Within each transmission scenario, the model estimated the final individual risk for acquiring monkeypox within each of the six sexual activity strata. The model also summarized the proportion of MPXV transmission that occurred via each partnership type. Finally, the reduction in the final proportion of MSM infected was estimated at baseline and under a scenario in which MSM decreased their one-time partnering by 40% 2.5 months after MPXV entered the population, which is similar to recent survey results (*2*). All simulations were conducted in R (version 4.2.0; R Foundation) using the EpiModel package (*8*).

MSM with more than one partner in the previous 3 weeks had 1.8–6.9 times the risk for acquiring monkeypox compared with those who only had one partner in the past 3 weeks, depending on the transmission scenario (Table). The higher transmission scenario resulted in larger differences in risk between men in higher and lower activity strata. For example, in the lower transmission scenario the men in the highest activity stratum had 3.6 times the risk for acquiring monkeypox compared with men who only had one partner in the past 3 weeks; in the higher transmission scenario these men had nearly seven times the risk for acquiring monkeypox. Activity strata with an average of fewer than one partner in the past 3 weeks led to decreased risk for acquiring monkeypox.

**TABLE:** Modeled mean number of partners, population size, and risk ratio for acquiring monkeypox among gay, bisexual, and other men who have sex with men, by level of sexual activity — United States, 2022*

Sexual activity stratum^†^	Mean no. and types^§^ of partners during time interval			% of population	RR (by transmission scenario)	
	Past yr	Past 3 wks				
	All types	All types	One-time only		Lower	Higher
1 (lowest)	1.8	0.8	0.0	19	0.6	0.5
2	1.8	0.8	0.0	19	0.7	0.5
3	4.0	0.9	0.1	19	0.9	0.9
4	4.0	1.0	0.2	19	1.0^¶^	1.0^¶^
5	14.7	1.5	0.7	19	1.8	2.3
6 (highest)	124.7	6.6	5.8	5	3.6	6.9

**Abbreviation:** RR = risk ratio.

* Contact data sources: https://linkinghub.elsevier.com/retrieve/pii/S1755436519301409; http://academic.oup.com/jid/article/214/12/1800/2632613; http://academic.oup.com/jid/article/214/12/1800/2632613

^†^ Based on rate of one-time partnership formation. MSM in stratum 1 have a 0.000 probability of having a one-time sexual partner on any given day, and MSM in stratum 6 have a 0.286 probability of having a one time-time sexual partner on any given day. Strata 2–5 have one-time partnership probabilities between these two endpoints on any given day.

^§^ Partnerships include main (assumed to last an average of 477 days), casual (assumed to last an average of 166 days), and one-time (assumed to last 1 day).

^¶^ Comparison group for RR calculation.

Modeled one-time partnerships had a disproportionate effect on transmission (Figure 1). Although one-time partnerships represented 3% of the total daily partnerships and 16% of the sexual contacts on any given day in the models, these partnerships accounted for 46%–54% of MPXV transmission, depending on the transmission scenario. In the lower transmission scenario, 54.0% of transmission occurred through one-time, 33.2% through casual, and 12.9% through main partnerships over the course of the outbreak. In the higher transmission scenario, 45.6% of transmission occurred through one-time, 38.8% through casual, and 15.6% through main partnerships over the course of the outbreak. In both lower and higher transmission scenarios, casual partnerships played a larger role in transmission than did main partnerships.

**FIGURE 1:**
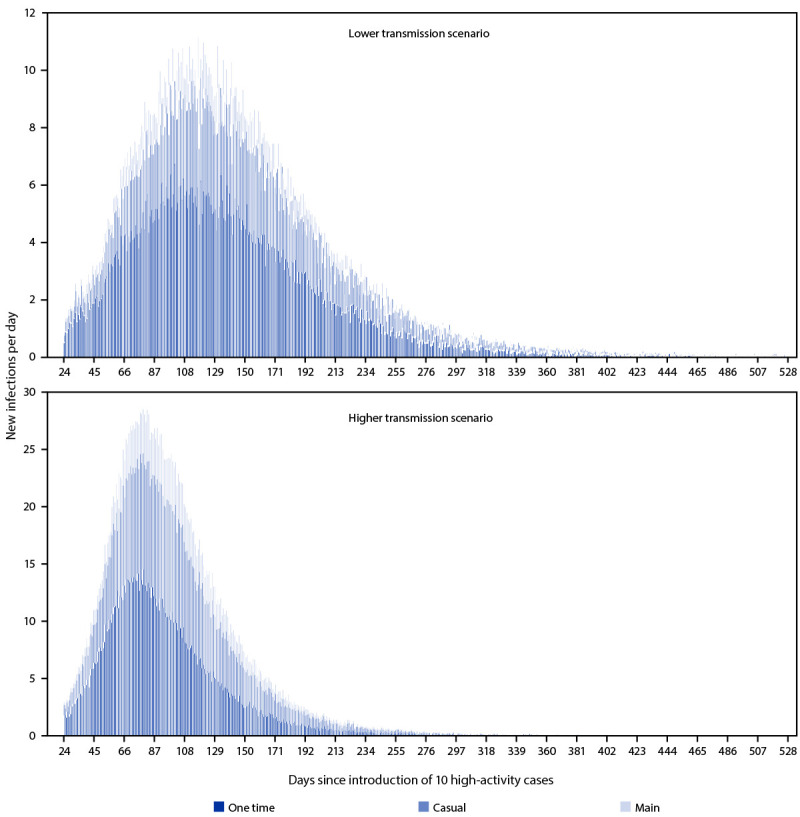
Modeled number* of new infections each day by lower^†^ and higher^§^ transmission scenarios and type of partnership over the course of a monkeypox outbreak among men who have sex with men, by time since importation of 10 high activity cases — United States, 2022 * Numbers presented are the mean number of infections across 60 stochastic trials in which no premature extinction occurred. ^†^ In the lower transmission scenario, it was assumed that there was a 60% probability of *Monkeypox virus* transmission per sex act: 54.0% of transmission occurred through one time, 33.2% through casual, and 12.9% through main partnerships over the course of the outbreak. ^§^ In the higher transmission scenario, it was assumed that there was a 90% probability of *Monkeypox virus t*ransmission per sex act: 45.6% of transmission occurred through one time, 38.8% through casual, and 15.6% through main partnerships over the course of the outbreak.

The model predicted that a 40% decrease in one-time partnerships would result in a 20%–31% reduction in the final percentage of MSM infected, depending on the transmission scenario (Figure 2), with larger impact in the lower transmission scenario. This impact could be stronger if combined with additional mitigation measures including vaccination or shorter time from symptom onset to testing and treatment. A decrease in one-time partnerships not only decreased the final percentage of MSM infected, but it also increased the number of days needed to reach a given level of infection in the population, allowing more time for vaccination efforts to reach susceptible persons. For example, reductions in one-time partnerships delayed the timing of 10% cumulative infection by approximately 150 days. Decreased one-time partnerships also led to fewer MSM being infected at any given time.

**FIGURE 2:**
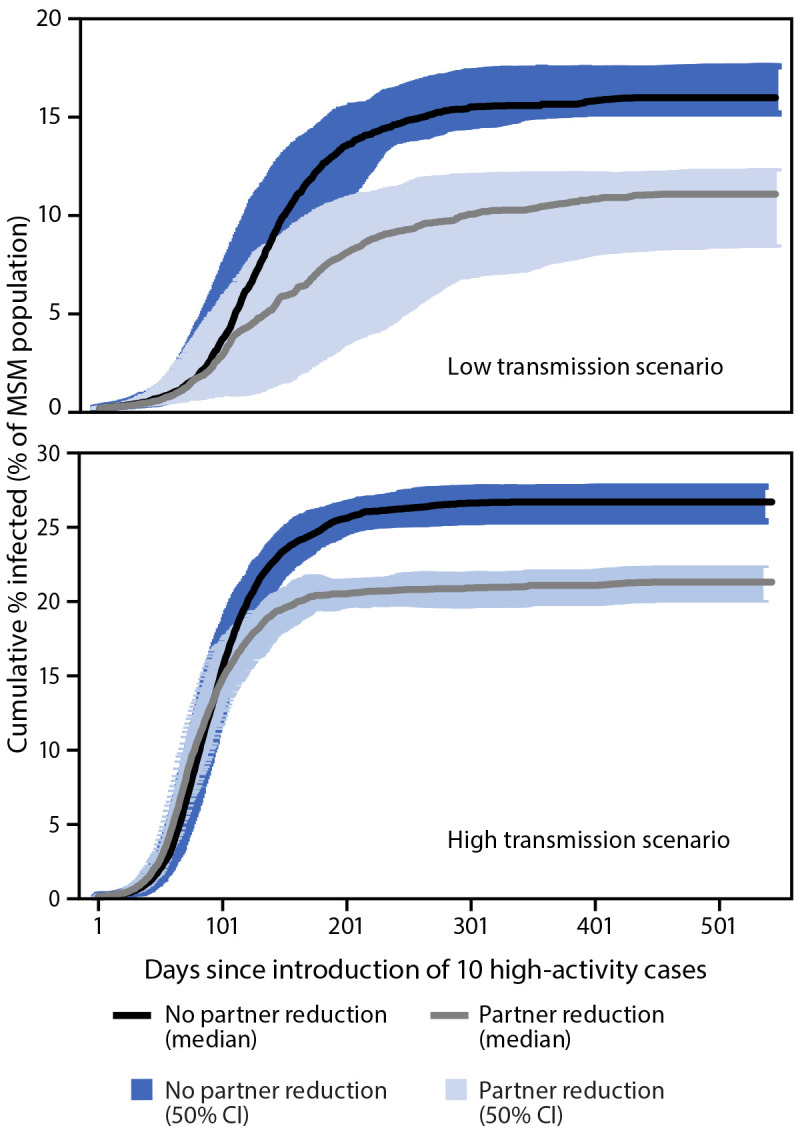
Modeled impact* of reduction in one-time sexual partners^†^ in a monkeypox outbreak among men who have sex with men with lower^§^ and higher¶ transmission scenarios, by days since importation of 10 high activity cases — United States, 2022 **Abbreviations:** CI = credibility interval; MSM = men who have sex with men. * The 50% CI were calculated from 60 stochastic trials in which no premature extinction occurred. ^†^ Represents a behavior change that assumes a 40% reduction in one-time partnership formation at day 75 of the outbreak. ^§^ In the lower transmission scenario, behavior change caused a 31% proportionate reduction in the final percentage infected. ^¶^ In the higher transmission scenario, behavior changed caused a 20% proportionate reduction in the final percentage infected.

## Discussion

This analysis illustrates that risk for MPXV acquisition varies widely among MSM according to the number of sexual partners a person has. In addition, one-time sexual partnerships are important contributors to the spread of MPXV through a sexual network. The model predicts that reductions in the number of one-time partnerships at a level already reported by MSM (*2*) might result in reductions in the final proportion of MSM infected and a slower-developing outbreak that would ultimately lead to fewer MSM infected at any given time. These changes might allow for additional time for other prevention measures, such as vaccination, to be more widely implemented and disseminated and reduce the impact on health care systems.

Although the importance of number of partners has been well established previously (*9*), the importance of one-time partnerships isn’t as widely understood. Having a large number of partners, as is facilitated by having many one-time partnerships, results in broad connectivity in a sexual network. This increases transmission of all sexually transmitted infections but is particularly important for an infection like monkeypox, which has a short, symptomatic contagious period.

The findings show that changes already being reported by MSM (*2*) can have important implications for the trajectory of the monkeypox outbreak. Current vaccination efforts are challenged by the speed with which the outbreak is spreading: the quicker the outbreak spreads, the faster persons become infected, resulting in insufficient time for many men who are susceptible and at risk to receive a vaccine. Because current vaccine supply is limited, measures that might delay the spread of MPXV, such as reduction in one-time partnerships, could be critical for broadening vaccine coverage and lowering the cumulative infection rate.

The findings in this report are subject to at least five limitations. First, data on MPXV transmission needed to develop these types of models are currently limited. The model included scenarios reflecting a lower and higher transmission potential of MPXV; however, the actual transmission potential of this outbreak might be outside the bounds considered. If MPXV were less intensely transmitted, a larger difference in infection risk between the highest and lowest activity strata and a larger impact of behavioral change would be anticipated. If MPXV were more intensely transmitted, a smaller difference in infection risk between the highest and lowest activity strata and a smaller impact of behavioral change would be anticipated. Second, superspreading events are not explicitly modeled. However, because persons in the highest activity stratum have approximately 100 partners per year, some of these one-time partnerships are occurring on the same day, which might adequately approximate a superspreading event. However, the model does not include specific events on a given date. Third, contact tracing is not modeled. Although contact tracing could help reduce infection levels, the exclusion of this mechanism is unlikely to change inferences related to number of partners or one-time partnerships. Fourth, regular reintroductions of MPXV infections are also not modeled. Ignoring regular importation (or exportation) of infections is also unlikely to affect inferences. Finally, the data used to characterize the sexual partnering behavior of MSM were collected among MSM aged <40 years at venues such as bars, bookstores, and other lesbian, gay, bisexual, and transgender–friendly places, and the data might not be representative of all MSM and might have oversampled MSM with more frequent sexual activity.

In the current outbreak, MPXV has been transmitted predominantly through close contact associated with sexual activity. Therefore, identifying factors that put persons at increased risk for acquiring and transmitting infection is critical to understanding transmission and tailoring mitigation strategies and control measures. These models show that personal decisions and public health interventions around one-time partnerships can have a substantial impact on reducing MPXV transmission. Changes in number of sex partners, and particularly changes in one-time partnerships, which are already being reported by MSM (*2*), have the potential to delay the spread of the outbreak. This could allow vaccination and implementation of other mitigation efforts to reach populations at high risk before they have been exposed to MPXV, and ultimately reduce MPXV transmission”

**Summary**

**What is already known about this topic?**

The 2022 monkeypox outbreak is associated with sexual and intimate contact. Survey data suggest that gay, bisexual, and other men who have sex with men (MSM), who have been disproportionately affected, are reducing one-time partnerships.

**What is added by this report?**

Modeling of sexual infection transmission between men indicates that one-time partnerships, which account for 3% of daily sexual partnerships and 16% of daily sex acts, account for approximately 50% of daily *Monkeypox virus* (MPXV) transmission. A 40% reduction in one-time partnerships might delay the spread of monkeypox and reduce the percentage of persons infected by 20% to 31%.

**What are the implications for public health practice?**

Reductions in one-time partnerships, already being reported by MSM, might significantly reduce MPXV transmission.

## Footnotes

* Modeling code is available for download. https://github.com/CDCgov/mpx_networkmodel_mmwr
